# Markers of T Cell Exhaustion and Senescence and Their Relationship to Plasma TGF-β Levels in Treated HIV+ Immune Non-responders

**DOI:** 10.3389/fimmu.2021.638010

**Published:** 2021-03-25

**Authors:** Carey L. Shive, Michael L. Freeman, Souheil-Antoine Younes, Corinne M. Kowal, David H. Canaday, Benigno Rodriguez, Michael M. Lederman, Donald D. Anthony

**Affiliations:** ^1^Louis Stokes Cleveland VA Medical Center, Cleveland, OH, United States; ^2^Center for AIDS Research, Department of Pathology, Case Western Reserve University, Cleveland, OH, United States; ^3^Center for AIDS Research, Division of Infectious Diseases and HIV Medicine, Department of Medicine, Case Western Reserve University/University Hospitals Cleveland Medical Center, Cleveland, OH, United States; ^4^MetroHealth Medical Center, Division of Rheumatic Disease, Case Western Reserve, Cleveland, OH, United States

**Keywords:** HIV+ immune non-responders, inflammation, senescence, exhaustion, T regulatory cells, TGF-β, IL-6, age

## Abstract

**Background:** Immune non-responders (INR) are HIV+, ART-controlled (>2 yrs) people who fail to reconstitute their CD4 T cell numbers. Systemic inflammation and markers of T cell senescence and exhaustion are observed in INR. This study aims to investigate T cell senescence and exhaustion and their possible association with soluble immune mediators and to understand the immune profile of HIV-infected INR. Selected participants were <50 years old to control for the confounder of older age.

**Methods:** Plasma levels of IL-6, IP10, sCD14, sCD163, and TGF-β and markers of T cell exhaustion (PD-1, TIGIT) and senescence (CD57, KLRG-1) were measured in ART-treated, HIV+ participants grouped by CD4 T cell counts (*n* = 63). Immune parameters were also measured in HIV-uninfected, age distribution-matched controls (HC; *n* = 30). Associations between T cell markers of exhaustion and senescence and plasma levels of immune mediators were examined by Spearman rank order statistics.

**Results:** Proportions of CD4 T cell subsets expressing markers of exhaustion (PD-1, TIGIT) and senescence (CD57, KLRG-1) were elevated in HIV+ participants. When comparing proportions between INR and IR, INR had higher proportions of CD4 memory PD-1+, EM CD57+, TEM TIGIT+ and CD8 EM and TEM TIGIT+ cells. Plasma levels of IL-6, IP10, and sCD14 were elevated during HIV infection. IP10 was higher in INR. Plasma TGF-β levels and CD4 cycling proportions of T regulatory cells were lower in INR. Proportions of CD4 T cells expressing TIGIT, PD-1, and CD57 positively correlated with plasma levels of IL-6. Plasma levels of TGF-β negatively correlated with proportions of TIGIT+ and PD-1+ T cell subsets.

**Conclusions:** INR have lower levels of TGF-β and decreased proportions of cycling CD4 T regulatory cells and may have difficulty controlling inflammation. IP10 is elevated in INR and is linked to higher proportions of T cell exhaustion and senescence seen in INR.

## Introduction

HIV infection, even when successfully controlled with antiretroviral therapy (ART), upsets the homeostasis of the immune system and is associated with increased morbidities such as cardiovascular disease (CVD) and cancer (1). While the life expectancy of a person infected with HIV has increased dramatically with the use of ART, it still remains shorter than in an HIV-uninfected person (2).

ART-treated HIV+ people exhibit continued elevation of systemic inflammation as indicated by plasma levels of soluble immune mediators that are associated with non-AIDS morbidities (3–5). Systemic inflammation is also prevalent in HIV-uninfected elderly, often referred to as “inflammaging” in those over 65 years old (6). Chronic elevated plasma levels of soluble immune mediators are also associated with morbidities and mortality in HIV-uninfected elderly (7, 8). Interleukin 6 (IL-6), tumor necrosis factor (TNF), and interleukin 1 beta (IL-1b) are among the earliest cytokines produced during an immune response, but when elevated levels of these cytokines persist they are linked to pathologies associated with chronic inflammation (9).

Immune senescence is also characteristic of both aging and chronic viral infection. T cell expression of inhibitory markers can identify exhausted (PD-1, TIGIT) or senescent (CD57, KLRG-1) T cells. Exhausted T cells undergo cell cycle arrest and lose polyfunctionality, including cytokine production (10). Programed cell death protein 1 (PD-1) is an inhibitory receptor associated with T cell exhaustion and is elevated in HIV disease (11, 12) and uninfected elderly (13, 14). T cell Immunoglobulin and ITIM Domain (TIGIT) is an inhibitory receptor on T cells that out-competes the activating receptor CD226 for its ligand-CD155. Increased frequencies of TIGIT+ effector CD8 T cells correlated with parameters of HIV disease progression (15) and TIGIT was upregulated on CD8 T cells in uninfected elderly adults (14). These two studies also found higher proportions of CD8 T cells co-expressing TIGIT and PD1. Like exhausted T cells, senescent T cells undergo cell cycle arrest; however, they continue to generate cytokines (10). Senescent cells produce large volumes of inflammatory cytokines such as IL-6 and IL-8 and are described as having a senescence-associated secretory phenotype (SASP) (16). T cell expression of CD57 is generally used to identify senescent T cells and HIV-specific CD8 T cells expressing CD57 lacked the ability to proliferate in response to antigen (17). Lastly, Killer cell lectin-like receptor subfamily G (KLRG-1) expresses an ITIM motif and after blocking its ligand, E-cadherin, KLRG-1 expressing CD8 T cells regained proliferative function (18). CD8 T cell expression of KLRG-1 is increased with age (19).

Cellular exhaustion or senescence often results after multiple rounds of proliferation which can lead to dysfunctional telomeres and the DNA damage response and cellular stress resulting in cell cycle arrest (20). Therefore, memory cells that have clonally expanded repeatedly are often the T cell maturation subsets that express markers of exhaustion and senescence.

Immune failure is a condition in HIV infection in which circulating CD4 T cell numbers fail to recover, or are very slow to recover, even when viremia is controlled by ART for >2 years. HIV+ people with immune failure are referred to as immune non-responders (INR), in contrast to immune responders (IR) who do recover CD4 T cell numbers. Immune failure is associated with increased non-AIDS morbidities (1), and INRs have both elevated systemic inflammation (21) and T cell exhaustion (22). We showed previously that INR express elevated levels of CD57 and PD-1 on their CD4 and CD8 T cells (22). In addition, we demonstrated that plasma levels of IL-6 and sCD14 were elevated in INR compared to plasma levels in IR (21). However, the INR in those studies were significantly older than the IR (21, 22). In the previous study INRs were more likely to be male and white, and they had lower CD4 nadir and were older at initiation of ART.

The objective of the current study was to examine the expression of T cell exhaustion and senescence markers and their possible associations with soluble immune mediators that have been associated with morbidity and mortality in HIV infection and aging; accounting for CD4 T cell counts and age. To determine if age may have influenced the results in the previous study of INR, we chose samples from participants that were <50 years old and would not be considered elderly (>65 years old).

HIV+ participants were on ART with controlled viremia for at least 2 years and were primarily male. The HIV+ participants were placed into three groups according to CD4 T cell status: INR (<350 cells/uL), intermediate (IT; 350–500 cells/uL), and IR (>500 cells/uL). HIV-uninfected, age distribution-matched controls (HC) were included for comparison. There was no significant difference in CD4 nadir or age at initiation of ART among the three groups of HIV+ participants.

This study was designed to examine systemic inflammation and markers of T cell exhaustion and senescence in ART-treated HIV+ INR and in age-matched uninfected controls. We found that T cell exhaustion and senescence negatively correlated with levels of TGF-β and positively correlated with plasma levels of IP10 and IL-6. Thus, we hypothesize that systemic inflammation contributes to the continuous activation of T cells resulting in T cell exhaustion and senescence.

## Methods

### Ethics Statement and Participant Samples

All subjects provided written informed consent in accordance with the Declaration of Helsinki. Participant studies were approved by the University Hospitals Cleveland Medical Center Institutional Review Board or the Cleveland VA Medical Center Institutional Review Board. Frozen peripheral blood mononuclear cell (PBMC) and plasma samples from ART-treated, HIV-infected patients were selected from the Case Western Reserve University Center for AIDS Research (CFAR) repository. Annually, patients in the HIV clinic at University Hospitals of Cleveland are asked if they would like to donate a blood sample to the CFAR repository. Patients who are interested are consented and PBMC and plasma samples are stored for future HIV research. For the current study, samples were requested from the repository from patients who had both PBMCs and plasma stored from the same date, were 50 years old or younger, were on HIV ART therapy for at least 2 years with controlled viremia and had similar CD4 nadir levels (no significant difference among INR, IT, IR groups). PBMC and plasma from healthy, HIV and HCV negative, consented participants 50 years old or younger were processed and stored as above.

### Flow Cytometry

T cell phenotype was assessed using the following fluorochrome conjugated monoclonal antibodies: anti-CD3 PerCP (clone SK7), anti-CD57 FITC (clone NK-1) (BD Biosciences, San Jose, CA), anti- KLRG-1 APC (clone 13F12F2) (eBiosciences, San Diego, CA), anti-CD4 Pacific Blue (clone RPA-T4), anti-CD8 APC-Cy7 (clone SK1), anti-CD45RA PE-Cy7 (clone HI100), anti-CD27 AlexaFluor 700 (clone M-T271), anti-PD-1/CD274 BV711 (clone EH12.2H7), anti-TIGIT PE (clone A15153G) (Biolegend, San Diego, CA). PBMCs were incubated with viability dye (LIVE/DEAD-Aqua, Invitrogen, Grand Island, NY) at room temperature for 20 min then washed. Monoclonal antibodies were added for 20 min in the dark at room temperature, washed, fixed in PBS containing 2% formaldehyde, and events were acquired on a BD LSRFortessa flow cytometer (Becton Dickinson, San Jose, CA). For detection of the intracellular proteins, Ki67 (anti-Ki67-PE, BD Biosciences, San Jose, CA) and FoxP3 (anti-FoxP3-Vio667/APC, Miltenyi Biotec, Auburn, CA) cells were surface stained as described above, fixed, and permeabilized using the reagents and instructions in the Treg Detection kit (Miltenyi Biotec, Auburn, CA). Data were analyzed using FACSDIVA, (version 6.2 BD Bioscience, San Diego CA) or FlowJo (version 10.5.0) software. Maturation subsets were determined based on expression of CD45RA and CD27; naïve = CD45RA+CD27+; central memory (CM) CD45RA-CD27+; effector memory (EM) CD45RA-CD27-; terminal effector memory (TEM) CD45RA+CD27-. If there were fewer than 100 events in any T cell memory subset, those data were excluded from analysis. The CD4 TEM T cell subset sometimes had fewer than 100 events and may have a lower “*n*” value.

### ELISA

Plasma IL-6 was measured by high sensitivity ELISA (Quantikine HS, R&D Systems, Minneapolis, MN), and soluble CD14 (sCD14), soluble CD163 (sCD163), Interferon gamma-induced protein 10 (IP10) or C-X-C motif chemokine 10 (CXCL10), and transforming growth factor-b1 (TGF-β1) were measured by ELISA (Quantikine, R&D Systems, Minneapolis, MN). Before measurement of TGF-β1, plasma was activated by 1N HCL as instructed in the manufacturer's protocol.

### Statistics

Dichotomous variables (gender and CMV sero-status) were examined using the Chi-squared test. Comparisons among three or more groups were performed with non-parametric Kruskal-Wallis tests. Comparisons between two unrelated groups were performed using non-parametric two-tailed Mann Whitney *U*-tests. Associations between continuous variables were explored by Spearman's rank order correlation coefficient. All statistics were performed using GraphPad version 6 and significance thresholds were set at *p*-values ≤ 0.05.

## Results

### Participant Characteristics

All HIV+ patients were ≤50 years old and had successful control of virus with ART for at least 2 years. Immune responder (IR) patients were more likely to have a protease inhibitor (PI) in their ART regimen (Table 1). No participants had a recognized viral illness at time of blood draw, no anti-neoplastic or immune modulatory treatment for cancer for at least 24 weeks, and no known immunological/inflammatory diseases (excepting HIV infection). There were no significant differences in age among the HC group and the INR, IT, and IR groups (Kruskal-Wallis *p* = 0.511; Table 1). There were no significant differences in CD4 nadir among the three HIV+ participant groups (Kruskal-Wallis *p* = 0.910; Table 1). Participants in all groups were primarily male. The HC participant group was primarily white (88%) and the HIV+ participant groups were about half white (INR 55%, IT 50%, and IR 42%). Nearly all HIV+ participants were serum antibody positive for cytomegalovirus (CMV) [INR 100% (23/23), IT 100% (14/14), IR 85% (22/26)] while 30% (9/30) of the HC participants were seropositive for CMV (Table 1).

**Table 1:** Participant characteristics.

	**HIV^**neg**^ HC**	**HIV^**pos**^ INR**	**HIV^**pos**^ IT**	**HIV^**pos**^ IR**	
***n*****=**	**30**	**23**	**14**	**26**	***P*****-value**
Median age (range)	41 (24–50)	43 (25–50)	44 (38–48)	41 (33–49)	*0.511
Median CD4 count (range)	NA	251 (80–347)	388 (352–491)	611 (503–1505)	*<0.0001
Median CD4+ Nadir (range)	NA	62 (0–255)	79 (0–203)	89 (3–249)	*0.91
Gender	76% male	77% male	100% male	75% male	**0.207
CMV % sero pos	30.0%	100%	100%	85%	**all grps = <0.0001 HIV+ grps = 0.048
Race	8% black	41% black	50% black	54% black	
	88%white	55% white	50% white	42% white	
	4% Other	4% Other		4% Other	
ART regimen	NA				
	NRTI + NNRTI	61%	57%	35%	
	NRTI + PI	30%^$^	36%	62%	
	NRTI + INSTI	4%	7%^∧^	4%	

*NA, not available; CMV, cytomegalovirus; NRTI, nucleoside reverse transcriptase inhibitors; NNRTI, non-nucleoside reverse transcriptase inhibitors; PI, protease inhibitor; INSTI, integrase strand transfer inhibitor*.

***HC** = uninfected age distribution-matched control (≤50 years old)*.

***INR** = immune non-responders; ART treated; <350 CD4 T cells/uL; <50 years old*.

***IT** = intermediate CD4 counts; ART treated; 350–500 CD4 T cells/uL; <50 years old*.

***IR** = immune responders; ART treated; >500 CD4 T cells/uL; <50 years old*.

*There was no significant difference in age among INR, IT, IR, or HC populations*.

* *Kruskal-Wallis*;

** *Chi Squared; one patient received PI only; one patient received PI also*.

### Proportions of T Cell Subsets and T Cell Subset Expression of Exhaustion and Senescence Markers in Treated, HIV-Infected Participants

**Figure 1:**
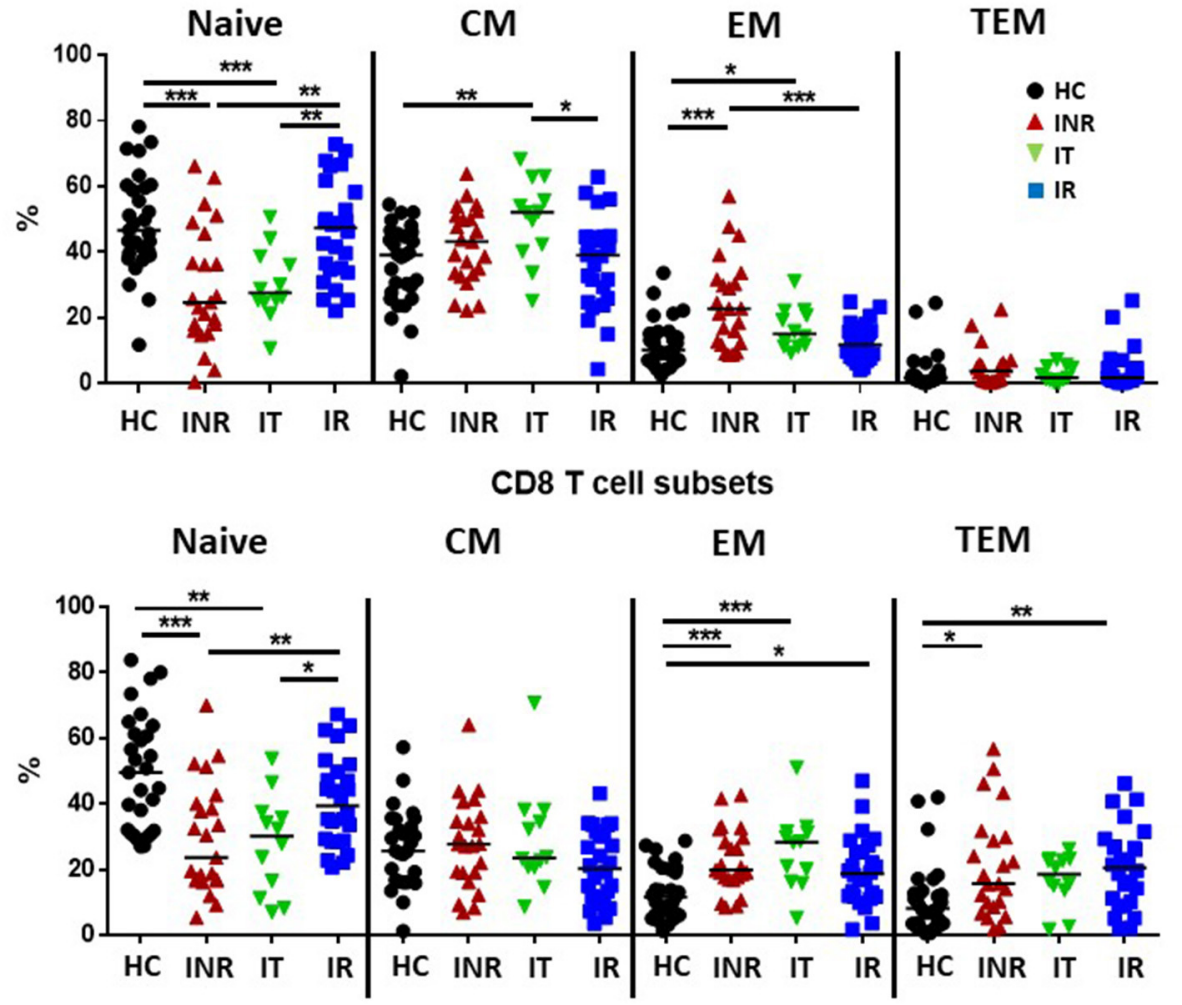
Proportions of T cell maturation subsets in healthy controls, immune non-responders, intermediate, and immune responders. Thawed PBMCs were stained with live/dead stain, for CD4 and CD8 T cell subsets, and for CD45RA and CD27 to determine maturation subsets (naïve= CD45RA+CD27+; central memory CM= CD45RA-CD27+; effector memory EM= CD45RA-CD27-; terminal effector memory TEM= CD45RA+CD27-). Comparisons between 2 groups were made using the non-parametric, unpaired Mann-Whitney test. *p = <0.05; **p = <0.01; ***p = <0.001.

Supplementary Figure 1A shows the gating strategy used to identify live CD3+ lymphocytes, CD4+ or CD8+ T cells, and naïve (CD45RA+CD27+), central memory [(CM) CD45RA-CD27+], effector memory [(EM) CD45RA-CD27-], and terminal effector memory [(TEM) CD45RA+CD27-] T cell maturation subsets. The proportions of naïve CD4 and CD8 T cells were lower in INR and IT compared to proportions in HC and IR, while proportions of effector memory T cells were higher in INR and IT than in HC. Effector memory CD8 T cell proportions were also elevated in IR compared to proportions in HC and effector memory CD4 T cell proportions were higher in in INR than in IR (Figure 1). Proportions of CD8 TEM T cells were elevated in both INR and IR compared to proportions in HC (Figure 1).

**Figure 2:**
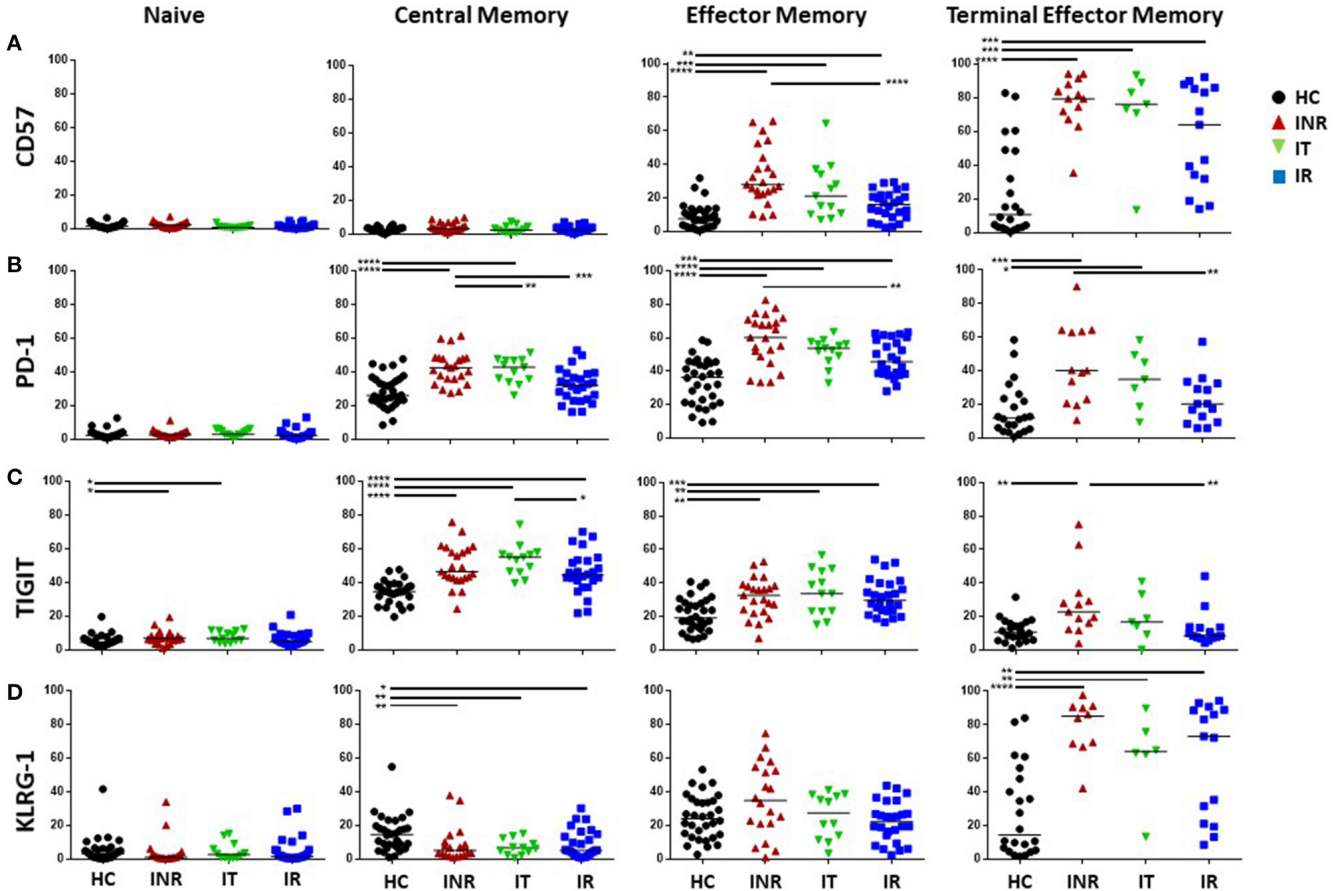
Proportions of T cell exhaustion and senescence markers on CD4 naïve, CM, EM, and TEM T cell subsets. Thawed PBMCs were stained with live/dead stain, for CD4 and CD8 T cell subsets, and for CD45RA and CD27 to determine maturation subsets (naïve = CD45RA+CD27+; central memory CM = CD45RA-CD27+; effector memory EM = CD45RA-CD27-; terminal effector memory TEM = CD45RA+CD27-). Live gated CD3+CD4+ T cells were assessed for the proportion of CD57 **(A)**, PD-1 **(B)**, TIGIT **(C)**, and KLRG-1 **(D)** within each maturation subset. Each symbol represents one participant, *black circles* = uninfected <50 years old; *red triangles* = ART treated HIV-infected INR CD4 <350/uL, <50 years old; *green inverted triangles* = ART treated HIV-infected IT CD4 350–500/uL, <50 years old; *blue squares* = ART treated HIV-infected IR CD4 >500/uL, <50 years old. Short black bars indicate median. Comparisons between 2 groups were made using the non-parametric, unpaired Mann-Whitney test. *p = <0.05; **p = <0.01; ***p = <0.001; ****p = <0.0001.

Examples of PD-1, KLRG-1, TIGIT (Supplementary Figure 1B), and CD57 (Supplementary Figure 1C) staining on CD4 and CD8 T cells, maturation subsets and isotype controls are shown in Supplementary Figures 1B,C. In general, the proportions of memory CD4 T cell subsets expressing CD57, PD-1, TIGIT, and KLRG-1 were elevated in HIV+ participants compared to proportions in uninfected participants (Figure 2). CD4 CM T cell proportions expressing TIGIT were higher in INR, IT and IR than proportions in HC. PD-1+ proportions of CD4 CM T cells were higher in INR and IT compared to proportions in HC. Proportions of KLRG-1+ CD4 CM T cells were higher in HC compared to those in the HIV-infected groups. Proportions of CD57+, PD-1+, and TIGIT+ CD4 EM T cells were higher in INR, IT, and IR compared to proportions in HC. CD4 TEM proportions of CD57+ and KLRG-1+ T cells were higher in INR, IT, and IR than in HC. Proportions of PD-1+ TEM T cells were higher in INR and IT compared to proportions in HC. Proportions of TIGIT+ TEM T cells were higher in INR when compared to proportions in HC.

In INR, the proportions of CD4 EM T cells expressing CD57 (Figure 2A) and PD-1 (Figure 2B) were elevated compared to the proportions in IR (Figures 2A,B). The proportions of TEM CD4 T cells expressing PD-1 and TIGIT were also elevated in INR compared to IR (Figures 2B,C).

**Figure 3:**
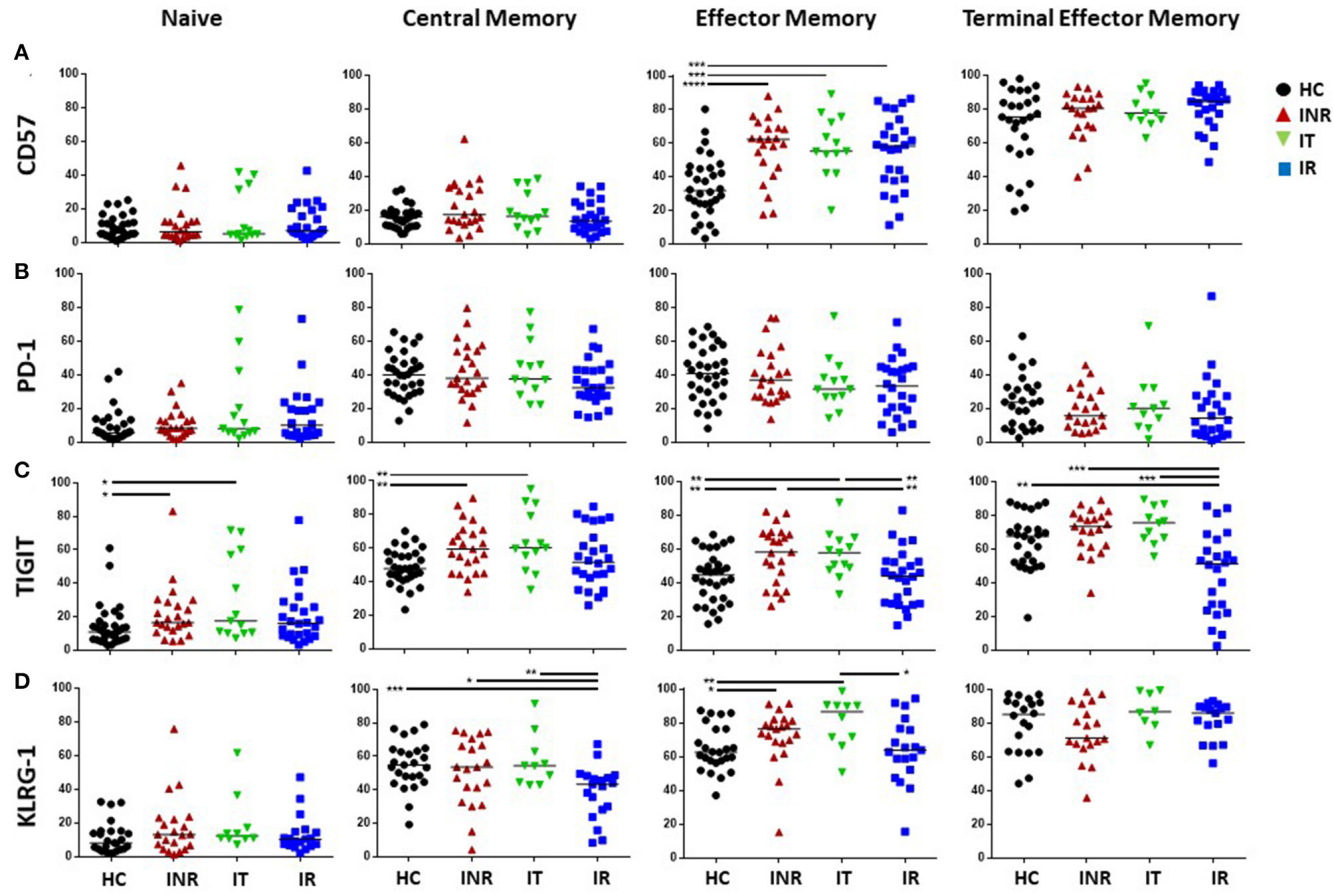
Proportions of T cell exhaustion and senescence markers on CD8 naïve, CM, EM, and TEM T cell subsets. Thawed PBMCs were stained with live/dead stain, for CD4 and CD8 T cell subsets, and for CD45RA and CD27 to determine maturation subsets (naïve = CD45RA+CD27+; central memory CM = CD45RA-CD27+; effector memory EM = CD45RA-CD27-; terminal effector memory TEM = CD45RA+CD27-). Live gated CD3+CD8+ T cells were assessed for the proportion of CD57 **(A)**, PD-1 **(B)**, TIGIT **(C)**, and KLRG-1 **(D)** within each maturation subset. Each symbol represents one participant, *black circles* = uninfected <50 years old; *red triangles* = ART treated HIV-infected INR CD4 <350/uL, <50 years old; *green inverted triangles* = ART treated HIV-infected IT CD4 350–500/uL, <50 years old; *blue squares* = ART treated HIV-infected IR CD4 >500/uL, <50 years old. Short black bars indicate median. Comparisons between 2 groups were made using the non-parametric, unpaired Mann-Whitney test. *p = <0.05; **p = <0.01; ***p = <0.001; ****p = <0.0001.

In general, the proportions of CD8 T cells expressing markers of exhaustion and senescence were higher than that on CD4 T cells, especially in CM and TEM subsets, but the number of significant differences among the groups were not as great (Figures 2, 3). CD8 CM T cell proportions expressing TIGIT were higher in INR and IT compared to proportions in HC and EM proportions of TIGIT+ and KLRG-1+ T cells were higher in INR and IT compared to CD8 EM T cell proportions in uninfected participants (Figure 3). CD8 EM proportions of CD57+ T cells were higher in INR, IT, and IR compared to HC (Figure 3). CD8 T cells from INR expressed higher proportions of TIGIT+ (EM and TEM) and KLRG-1+ (CM) T cells than did memory CD8 T cells from IR. Surprisingly, the proportions of KLRG-1+ CM CD8 T cells and TIGIT+ TEM T cells were lower in IR than proportions in HC (Figures 3C,D).

### Plasma Levels of IL-6, IP10 (CXCL10), sCD14, and sCD163 in Treated, HIV+ Participants

**Figure 4:**
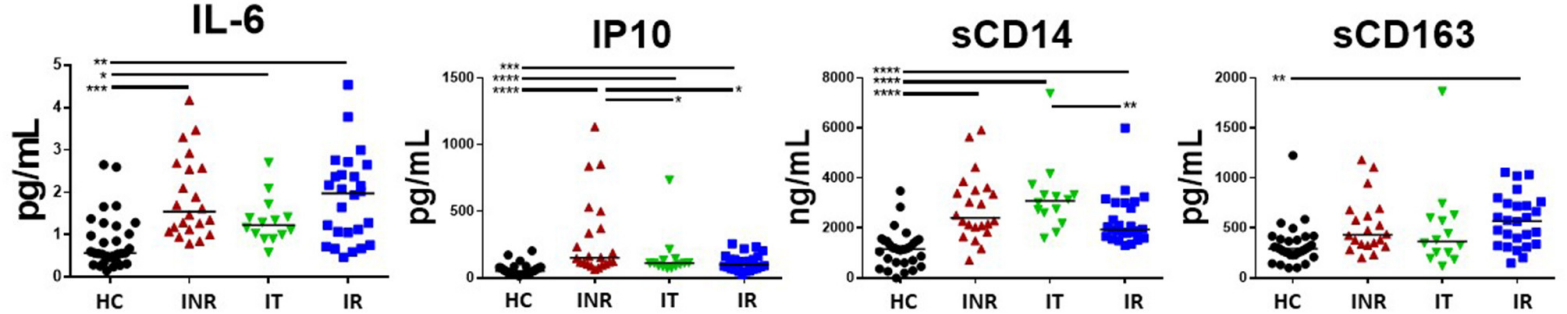
Plasma levels of IL-6, IP10 (CXCL10), sCD14, and sCD163 in treated, HIV+ participants. Plasma was measured by ELISA for IP10 (CXCL10), sCD14, sCD163, and IL-6 (high sensitivity) following the manufacture's protocol. Each symbol represents one participant, *black circles* = uninfected age-matched controls (HC) <50 years old; *red triangles* = ART treated HIV-infected INR CD4 <350/uL, <50 years old; *green inverted triangles* = ART treated HIV-infected IT CD4 350–500/uL, <50 years old; *blue squares* = ART treated HIV-infected IR CD4 >500/uL, <50 years old. Black bars indicate median. Comparisons between 2 groups were made using a Mann-Whitney *U*-test. *p = <0.05; **p = <0.01; ***p = <0.001; ****p = <0.0001.

Plasma IL-6 levels have been strongly associated with morbidities in HIV+ patients (3, 4) and were previously found to be elevated in the plasma of INR (21). However, in this cohort of relatively young (<50 years old) HIV+ patients, levels of IL-6 were not elevated in the plasma from INR when compared to plasma levels in the IR patients (Figure 4). Plasma levels of IL-6 were elevated in all HIV+ participant groups when compared to IL-6 levels in the HC group (Figure 4). Plasma IP10 levels were significantly elevated in the INR as compared to levels in IR and IT, and in all HIV+ groups compared to plasma levels in HC (Figure 4). There was a trend to higher plasma levels of sCD14 in HIV+ participants with lower CD4 counts, but this was only significant in the IT group compared to the IR group (Figure 4). Plasma levels of sCD14 were higher in HIV+ participant regardless of CD4 counts when compared to plasma levels in HC (Figure 4). Lastly, in this study, the only statistically significant difference in plasma sCD163 levels was an increase in plasma sCD163 in the IR compared to the plasma levels in the HC (Figure 4).

### Plasma Levels of TGF-β and Proportions of Cycling CD4 Cells That Are T Regulatory Cells in Treated HIV+ Participants

**Figure 5:**
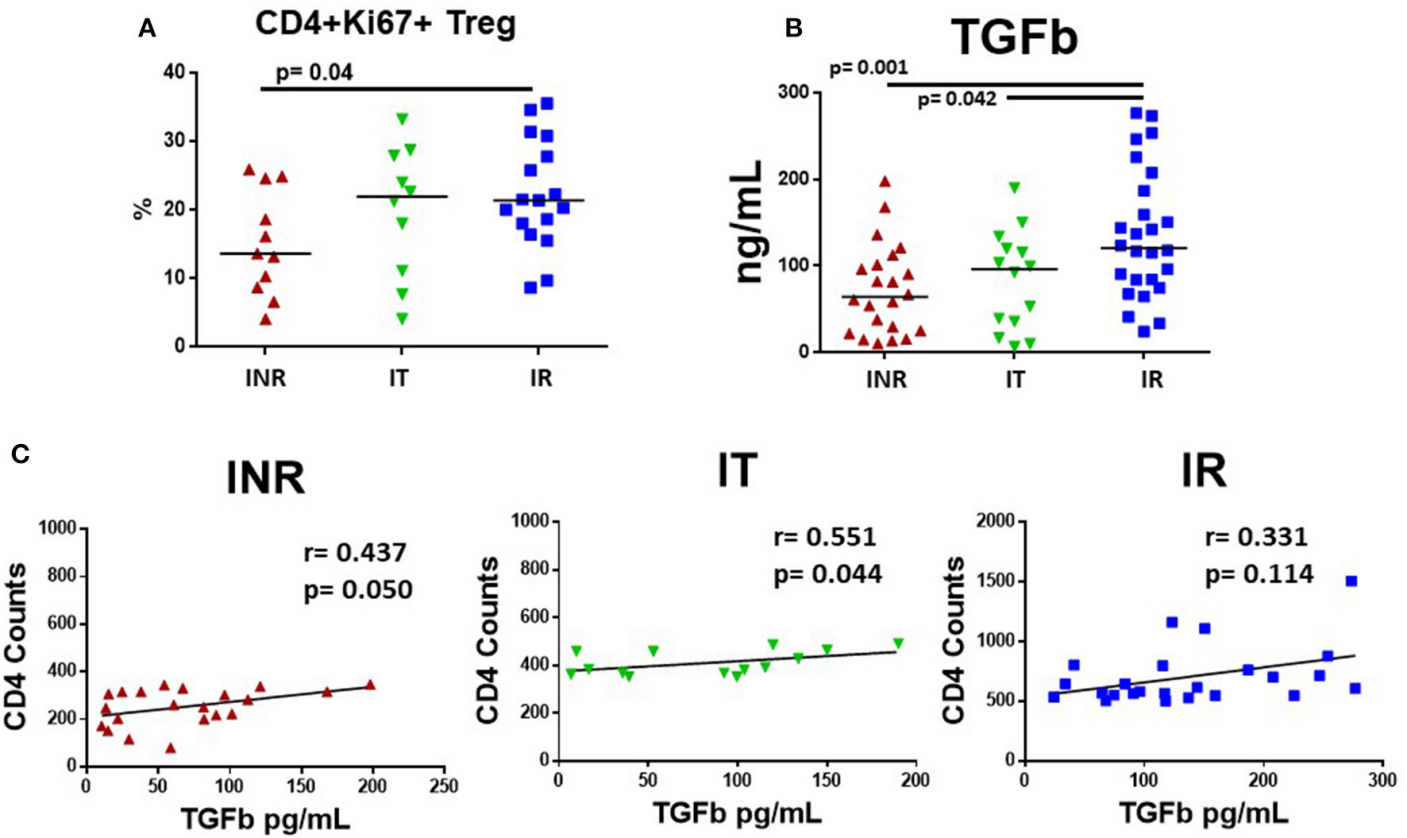
Plasma levels of TGF-β and proportions of cycling memory CD4+ cells that are T regulatory cells in HIV+, treated participants. PBMCs were stained for Tregs using Miltenyi Biotec's Treg Detection kit and plasma was measured for TGF-β using R&D System ELISA kit. Each symbol represents one subject, *red triangles* = INR, CD4 <350/uL, <50 years old; *green inverted triangles* = IT CD4 350–500/uL, <50 years old; *blue squares* = IR CD4 >500/uL, <50 years old. **(A)** Proportions of CD3+CD4+CD45RA-Ki67+ cells that were CD127-CD25+ and FoxP3+, identified as Tregs are shown. **(B)** TGF-β was activated with 1N HCL and measured following manufacture's protocol; plasma levels are shown. Comparisons between 2 groups were made using a Mann-Whitney *U*-test. **(C)** Plasma levels of TGF-β were compared to CD4 T cell counts in treated, HIV-infected INR, IT, and IR participants using a rank order Spearman's analysis. Significance thresholds were set at *p*-values equal to or <0.05.

We recently examined cycling CD4 T cells in INR and found that although cycling CD4 T cells were enriched for T regulatory cells (Tregs), frequencies of Tregs among cycling cells were lower in INR than in IR (23). Here, we examined the proportion of cycling (Ki67+) CD4 T cells that were Tregs in our three groups of HIV+ participants. Consistent with our previous report, we found that the proportion of CD45RA^neg^ CD4+ Ki67+ T cells that were Tregs (CD4+ CD45RA- CD25+ CD127- FoxP3+) were significantly lower in INR (Figure 5A) than in the IR group. TGF-β promotes the development of Tregs (24). Therefore, we examined TGF-β in the HIV+ participants and found that plasma TGF-β levels were significantly lower in the INR and IT groups when compared to plasma levels in the IR group (Figure 5B). Due to differences in collection protocols, plasma levels of TGF-β in HC were not directly comparable to HIV+ participant groups, and so have been excluded from these analyses. In addition, there was a positive correlation between CD4 T cell counts and plasma levels of TGF-β in INR (*r* = 0.437, *p* = 0.050) and IT (*r* = 0.551, *p* = 0.044) but not IR participants (*r* = 0.331, *p* = 0.114) (Figure 5C).

### The Association of Plasma Levels of IL-6, IP10, TGF-β, and sCD14 With T Cell Markers of Exhaustion and Senescence in Treated, HIV+ Participants

**Figure 6:**
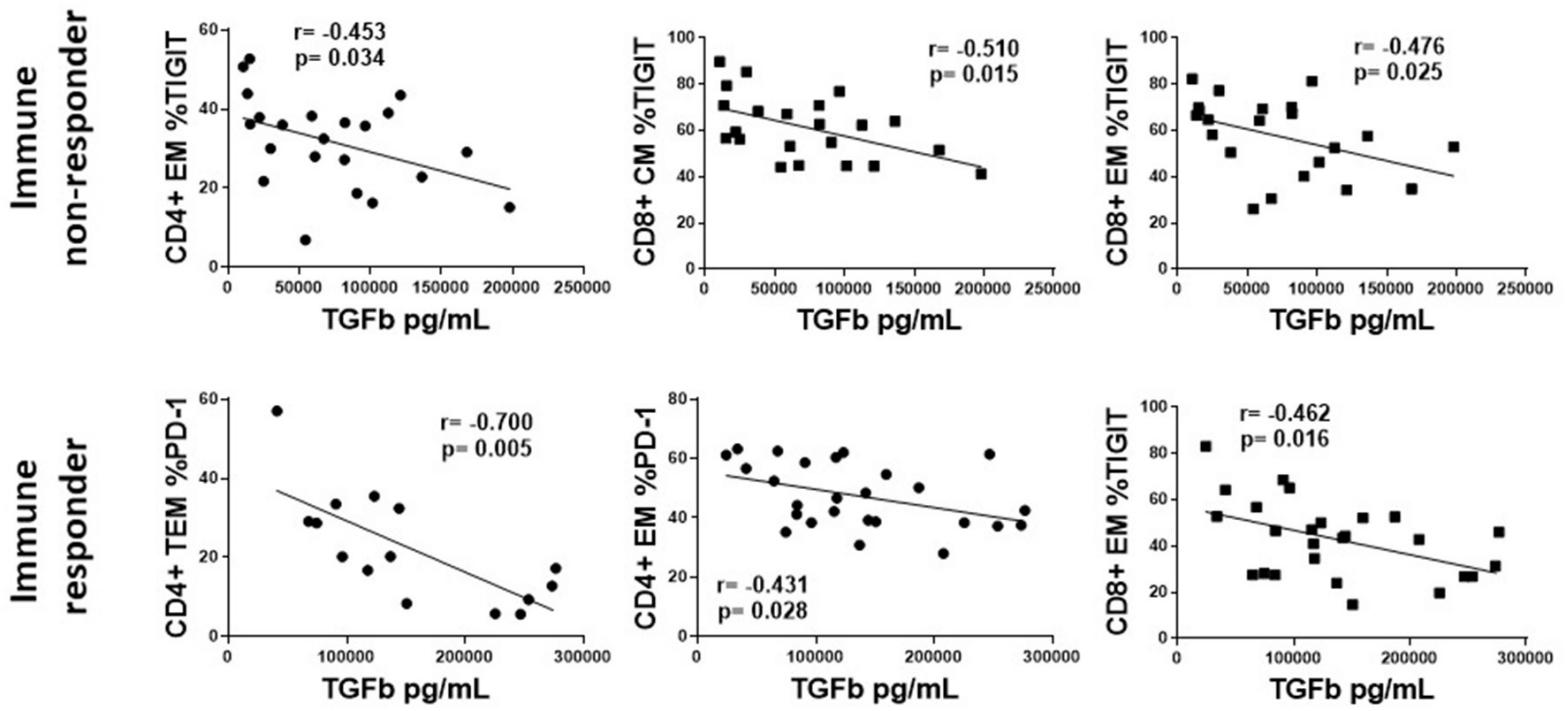
The association of plasma levels of TGF-β with T cell markers of exhaustion and senescence in treated, HIV-infected immune non-responders and responders. Representative correlations from Supplemental Table 1 are show. Top row, immune non-responders (INR); Plasma levels of TGF-β (x-axis) vs. CD4+ EM proportion of TIGIT, or CD8+ CM and EM proportions of TIGIT (y-axis). Bottom row, immune responders (IR); Plasma levels of TGF-β (x-axis) vs. CD4+ EM and TEM proportion of PD-1, or CD8+ EM proportions of TIGIT. Correlations were calculated using a Spearman's rank order analysis; *p* = <0.05 considered statistically significant.

Next, we examined the correlation of plasma levels of IL-6, IP10, TGF-β, sCD14, and sCD163 with the proportions of markers of T cell exhaustion (PD-1, TIGIT) and senescence (CD57, KLRG-1) expressed on T cell maturation subsets in HIV+ participants. Only those associations that were significant are shown in Supplementary Table 1. Figure 6 shows representative graphs of the association between plasma levels of TGF-β and TIGIT+ T cell subset proportions in INR and plasma TGF-β levels and T cell subset proportions of TIGIT+ and PD-1+ cells in IR. Plasma levels of TGF-β negatively correlated with proportions TIGIT+ (CD4 EM, CD8 CM, EM) T cells in INR and proportions of TIGIT+ (CD8 EM, naïve) and PD-1+ (CD4 EM, TEM) T cells in IR (Supplementary Table 1). Proportions of naïve KLRG-1+ T cells also negatively correlated with plasma levels of TGF-β in INR and IR (Supplementary Table 1). There was a positive correlation between plasma levels of TGF-β and proportions of CD4 EM CD57 in INR (Supplementary Table 1).

**Figure 7:**
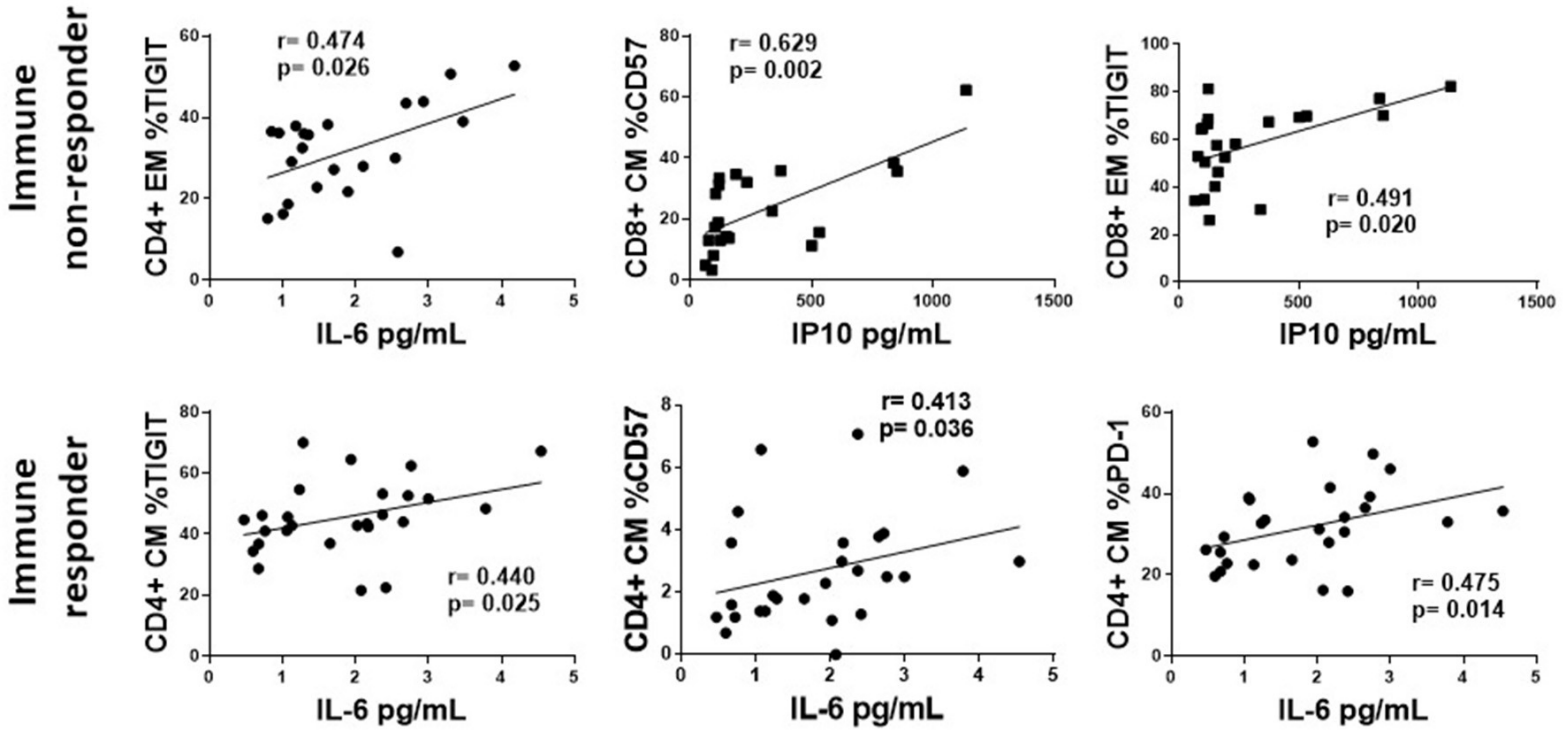
The association of plasma levels of IP10 and IL-6 with T cell exhaustion and senescence. Representative correlations from Supplemental Table 1 are show. Top row, immune non-responders (INR); Plasma levels of IL-6 or IP10 (x-axis) vs. CD4+ EM proportion of TIGIT, or CD8+ CM CD57 and EM proportions of TIGIT (y-axis, respectively). Bottom row, immune responders (IR); Plasma levels of IL-6 (x-axis) vs. CD4+ CM proportion of TIGIT, CD57, and PD-1. Correlations were calculated using a Spearman's rank order analysis; *p* = <0.05 considered statistically significant.

The associations of plasma levels of TGF-β with markers of T cell exhaustion were mostly negative associations, however the associations between plasma levels of IP10 and IL-6 and T cell markers of exhaustion and senescence were primarily positive (Supplementary Table 1). Figure 7 shows representative graphs of the positive association between plasma levels of IL-6 and proportions of CD4 EM TIGIT+ T cells and plasma levels of IP10 and proportions of CD8 EM TIGIT and CM CD57 in INR (upper panel). Plasma levels of IP10 also positively associated with proportions of CD4 EM PD-1 and CD8 CM PD-1+ cells in INR (Supplementary Table 1). The lower panels show the positive association between plasma levels of IL-6 with CD4 CM proportions of TIGIT+, PD-1+ and CD57+ cells in IR. There were no significant associations between plasma levels of IP10 and T cell markers of exhaustion or senescence in IR (Supplementary Table 1). There were few significant associations between sCD163 and markers of exhaustion and senescence (data not shown). Interestingly, in INR there was a negative association between plasma levels of sCD14 and proportions of CD8 T cell subsets expressing markers of exhaustion and senescence (Supplementary Table 1).

## Discussion

Our previous studies found elevated plasma levels of IL-6 and sCD14 (21) and increased T cell expression of CD57 and PD-1 (22) in INR. However, in those studies the INR were significantly older than the IR, and both INR and IR were significantly older than the uninfected controls (21, 22). IL-6 and sCD14 are elevated in HIV-uninfected elderly and are associated with morbidity and mortality (7, 8). In addition, T cell expression of PD-1 (13), TIGIT (14), and KLRG-1 (19) were elevated in HIV-uninfected elderly. Therefore, in the current study we chose treated, HIV-infected participants that were <50 years old and a group of uninfected, age distribution-matched healthy controls. All HIV+ participants had controlled viremia for at least 2 years. They were divided into groups based on CD4 T cell counts as described above. The other objective of the current study was to examine the expression of T cell exhaustion and senescence markers and their possible associations with soluble immune mediators in treated HIV infection.

As mentioned, markers of exhaustion and senescence are most often found on memory T cells that have undergone multiple rounds of replication and activation. Proportions of memory T cells are elevated during aging and in chronic viral infections and our data confirm these findings. In this study we did not calculate absolute naïve T cell counts.

Proportions of PD-1 expressing exhausted T cells are elevated in HIV disease (11, 12) and may be related to the ability to recover CD4 T cells after control of virus with ART (25). Examination of HIV-specific CD8 T cells found that many expressed CD57 and lacked the ability to proliferate in response to antigen (17) and T cell expression of CD57 may be associated with the inability to reconstitute CD4 T cells despite ART control of virus (26). In the current study we saw increased proportions of CD4 EM T cells expressing PD-1, TIGIT and CD57 and increased CD8 EM proportions of CD57, TIGIT and KLRG-1 in treated, HIV+ participants when compared to proportions expressed in uninfected controls. We also found that proportions of CD4 EM T cells expressing PD-1 and CD57 were elevated in INR when compared to proportions expressed in IR. Although the proportions of memory T cells were elevated in INR, we calculated the proportion of CD57, PD-1, TIGIT, and KLRG-1 expressing cells within each T cell maturation subset, therefore expression level should be independent of the proportion of the maturation subset.

We also examined T cell expression of the inhibitory markers, KLRG-1 and TIGIT. Increased frequencies of TIGIT expressing CD8 T cells correlated with parameters of HIV disease progression (15) and TIGIT was also upregulated on CD8 T cells in uninfected elderly adults (14). We found elevated proportions of TIGIT expressing CD4 (CM, EM, TEM) and CD8 (CM, EM) T cells in treated HIV+ participants compared to proportions in HC and proportions of TIGIT+ CD4 and CD8 TEM T cells tended to be higher in INR.

We found it interesting that proportions of TIGIT+ naive CD4 and CD8 T cells were elevated in INR and IT compared to proportions in HC. In a study of elderly patients, TIGIT was also elevated in naïve (CD45RA+CCR7+) CD8 T cells (14). The expression of exhaustion and senescence markers on naïve T cells from elderly participants may reflect a population of CD8 T cells that are phenotypically naïve (CD45RA+, CCR7+, CD27+ CD95-) but are actually antigen-experienced (13, 27). This population of memory T cells with a naïve phenotype (T_MNP_) may be present in INR as well. Our future studies in INR will examine this possibility in more detail.

Circulating inflammatory cytokines like IL-6 and IP10 are indicators of immune cell activation. Circulating soluble receptors are also an indication of innate immune cell activation. Activation of monocytes and macrophages causes the release of soluble forms of the surface receptors CD14 and CD163 (28, 29). In the current study, in which all HIV+ participant groups were similarly aged, plasma levels of IL-6 were higher in the HIV+ participants compared to plasma levels in HC, but among HIV+ donors, we observed no significant difference between INR and IR. IP10 is induced by Type I and Type II IFNs and in acute HIV infection, IP10 plasma levels were predictive of rapid disease progression and were negatively associated with CD4 T cell number set point (30). In the current study, plasma levels of IP10 were significantly higher in INR than in all other groups.

One factor that may contribute to systemic inflammation and T cell exhaustion and senescence observed in the current study is chronic infection with CMV. Indeed, CMV seropositivity is associated with immunosenescence (31) and our studies in HIV infection demonstrated that CMV coinfection is associated with elevated CD57 expression on both CD4 and CD8 memory T cells in treated HIV infection (32, 33). Another study found higher proportions of CD8+ CD28- T cells expressing CD57 in uninfected CMV seropositive participants compared to proportions in CMV seronegative participants (34). Proportions of CD8+CD28-T cells expressing CD57 were also associated with age (34). During primary CMV infection, IL-6 and IP10 are induced, and IP10 persists even during CMV latency (35). Previously, we found that plasma IP10 levels were significantly elevated in treated HIV+ CMV-seropositive donors compared to levels in treated HIV+ CMV-seronegative donors (36). It is possible that immune mechanisms that induce IP10, such as Type I or Type II IFNs, may arise early in HIV infection or as a result of CMV infection and persist after virus suppression by ART. Sorting out the immune manifestations of CMV infection is difficult because in immune competent hosts, infection is largely asymptomatic, and most HIV+ persons are CMV seropositive (93.7% in this cohort) (37). One limitation of the current study was that it was not powered to examine the effects of CMV infection on inflammation or T cell exhaustion/senescence, as nearly all of our HIV+ participants were CMV-seropositive, but only 30% of HC were CMV-seropositive. When we compared results from the CMV+ (*n* = 9) and CMV- (*n* = 21) uninfected healthy controls in the current study, we observed higher proportions of CD57+, PD-1+ and KLRG-1+ CD4 (EM and TEM) T cells in CMV seropositive HIV-uninfected controls compared to proportions in CMV seronegative HIV-uninfected controls. We did not see any significant difference in CD8 subset expression of markers of T cell exhaustion or senescence, nor did we see any significant difference in plasma levels of IL-6, IP10, sCD14, or sCD163 when results among CMV seropositive and seronegative controls were compared.

We hypothesize that systemic inflammation contributes to the continuous activation and turnover of T cells resulting in T cell exhaustion and senescence in INR. We saw a significant positive correlation between plasma levels of IL-6 and T cell expression of exhaustion and senescence markers in both INR and IR and between plasm levels of IP10 and T cell expression of exhaustion and senescence markers in INR. Although associations do not confirm causality or directionality, these data are consistent with the interpretation that systemic immune mediators are related to T cell exhaustion and senescence. Importantly, we have evidence suggesting causality in our earlier study in which we showed that *in vitro* stimulation of healthy PBMCs for 7 days with IL-6 or IL-1b can induce the expression of PD-1 and CD57 on T cells (22).

Perhaps even more striking in the current study was the negative association of plasma levels of TGF-β and expression of T cell markers of exhaustion and senescence in the INR and IR. TGF-β is an important anti-inflammatory cytokine and promotes the development of T regulatory cells (24). In our previous study examining INR, we paradoxically found higher levels of cycling (Ki67+) CD4 T cells in INR, even though they sustain low CD4 T cell numbers (21). In our more recent study that examined this finding in more detail, we found that many of the cycling CD4 T cells were Tregs regardless of the extent of CD4 T cell recovery. Previously, when we examined the cycling CD4 T cells, we found that cycling cells from INR had lower frequencies of CD4 Tregs overall, and the Tregs that remained had evidence of mitochondrial dysfunction (23). We also found that the cycling CD4 T cells in INR had transcriptomic profiles consistent with decreased TGF-β signaling and increased apoptosis signaling (23). Here, we found significantly lower plasma levels of TGF-β and cycling CD4+ T cells that were Tregs, as well as higher levels of T cell exhaustion and senescence in INR than among IR. We propose that there are mechanistic links among low TGF-β levels, reduced CD4 T cell recovery, impaired Treg functionality, and dysregulated T cell phenotypes.

In summary, treated, HIV+ participants ≤50 years old showed greater expression of T cell exhaustion (CD4 EM PD-1 and TIGIT; CD8 EM TIGIT) and senescence (CD4 and CD8 EM CD57) markers and higher plasma levels of IL-6, IP10 and sCD14 than did age matched HC. INR in particular, generally had greater T cell exhaustion (CD4 PD-1; CD8 TIGIT) and senescence (CD4 EM CD57) and elevated plasma levels of IP10. There was a positive correlation between plasma levels of IL-6 and CD4 T cell exhaustion [in INR and IT (EM TIGIT); in IR (CM TIGIT and PD-1)] and senescence [in IR (CM CD57)]. There was also a positive correlation between plasma IP10 and T cell exhaustion in INR (CD4 EM PD-1, CD8 CM PD-1, CD8 EM TIGIT) and T cell senescence in INR (CD8 CM CD57). Plasma levels of TGF-β and proportions of cycling CD4+ T cells that were Tregs were significantly lower in INR compared to levels in IR. Lastly, plasma levels of TGF-β negatively correlated with markers of T cell exhaustion in INR (CD4 EM TIGIT, CD8 CM EM TIGIT) and IR (CD4 EM TEM PD-1, CD8 EM TIGIT).

In conclusion, we hypothesize that a “tug-of-war” exists between inflammation and the associated T cell exhaustion and senescence on one side, and anti-inflammatory TGF-β levels and Tregs on the other. Our data support a model whereby INR have dysfunctional CD4 T cell cycling (23), lower levels of TGF-β, and decreased generation of Tregs, and therefore, may have difficulty controlling inflammation. Elevated plasma levels of IP10 and IL-6 were positively associated with T cell expression of exhaustion and senescence markers in INR in this study. Our previous *in vitro* studies showed that IL-6 and IL-1b can induce the upregulation of PD-1 and CD57 on T cells (22). Whether these effects contribute to or are a result of immune failure is an open question, and is the focus of current research in our laboratories. Future studies will examine this model in other settings of lymphopenia and in the HIV-uninfected elderly.

## Data Availability Statement

The raw data supporting the conclusions of this article will be made available by the authors, without undue reservation.

## Ethics Statement

The studies involving human participants were reviewed and approved by the University Hospitals Cleveland Medical Center Institutional Review Board or the Cleveland VA Medical Center Institutional Review Board. The patients/participants provided their written informed consent to participate in this study.

## Author Contributions

CS conceived study design, performed and analyzed experiments, and wrote manuscript. MF and S-AY contributed to experiment analysis and manuscript preparation. CK performed and analyzed experiments and CK, DC, and BR provided patient samples. DC, BR, ML, and DA contributed to study design and manuscript preparation. All authors reviewed and approved manuscript.

## Conflict of Interest

The authors declare that the research was conducted in the absence of any commercial or financial relationships that could be construed as a potential conflict of interest.

## Supplementary Material

Supplementary Figure 1 Gating strategy. **(A)** Shows the gating strategy for CD4 and CD8 T cell maturation subsets in two participants (INR and IR). After singlet gating, lymphocytes were gated on a FSC/SSC plot. Live cells were gated based on the negative staining of InVitrogen live/dead aqua stain, then live cells expressing CD3 were gated. CD3+ T cells were then gated for CD4 or CD8 expression. For each subset (CD4 or CD8) CD45RA vs. CD27 was examine and a quadrant gate was used to determine naïve (CD45RA+CD27+), central memory (CD45RA-CD27+), effector memory (CD45RA-CD27-), and terminal effector memory (CD45RA+CD27-) subsets. **(B)** Shows PD-1, CD57, KLRG-1, and TIGIT staining from one participant. The top row shows isotype staining in CD4 (blue) or CD8 (purple) T cells. The center row show PD-1, CD57, KLRG-1, and TIGIT staining in CD4 (blue) or CD8 (purple) T cells. The bottom row shows the PD-1, CD57, KLRG-1, and TIGIT staining in overlapping histographs of maturation subset in CD4 or CD8 T cells. The participant in **(B)** did not show positive CD57 staining, therefore we included a different participant who did show positive CD57 staining in **(C)**.

Supplementary Table 1 The association of plasma levels of IL-6, IP10, TGF-β, and sCD14 with markers of T cell exhaustion and senescence. The correlation of plasma levels of IL-6, IP10, TGFb, sCD14, and sCD163 were compared to the proportions of CD4 and CD8 T cell maturation subsets expressing CD57, PD-1, TIGIT, and KLRG-1 in treated, HIV+ INR, IT, and IR using Spearman's rank order analysis. Only correlations that were significant are shown in table. *P* and *r* values are shown for each significant correlation; *p* = <0.05 considered statistically significant. Gray boxes are significant negative correlations; white boxes are significant positive correlations.
