# Supplementary material for: Markers of T Cell Exhaustion and Senescence and Their Relationship to Plasma TGF-β Levels in Treated HIV+ Immune Non-responders
Source: Front Immunol. 2021 Mar 25;12:638010. doi: 10.3389/fimmu.2021.638010 (PMC8044907; doi:10.3389/fimmu.2021.638010)
Supplement: Supplementary Figure 1 — Gating strategy. (A) Shows the gating strategy for CD4 and CD8 T cell maturation subsets in two participants (INR and IR). After singlet gating, lymphocytes were gated on a FSC/SSC plot. Live cells were gated based on the negative staining of InVitrogen live/dead aqua stain, then live cells expressing CD3 were gated. CD3+ T cells were then gated for CD4 or CD8 expression. For each subset (CD4 or CD8) CD45RA vs. CD27 was examine and a quadrant gate was used to determine naïve (CD45RA+CD27+), central memory (CD45RA-CD27+), effector memory (CD45RA-CD27-), and terminal effector memory (CD45RA+CD27-) subsets. (B) Shows PD-1, CD57, KLRG-1, and TIGIT staining from one participant. The top row shows isotype staining in CD4 (blue) or CD8 (purple) T cells. The center row show PD-1, CD57, KLRG-1, and TIGIT staining in CD4 (blue) or CD8 (purple) T cells. The bottom row shows the PD-1, CD57, KLRG-1, and TIGIT staining in overlapping histographs of maturation subset in CD4 or CD8 T cells. The participant in (B) did not show positive CD57 staining, therefore we included a different participant who did show positive CD57 staining in (C). [file Presentation_1.PPTX]

## Slide 1
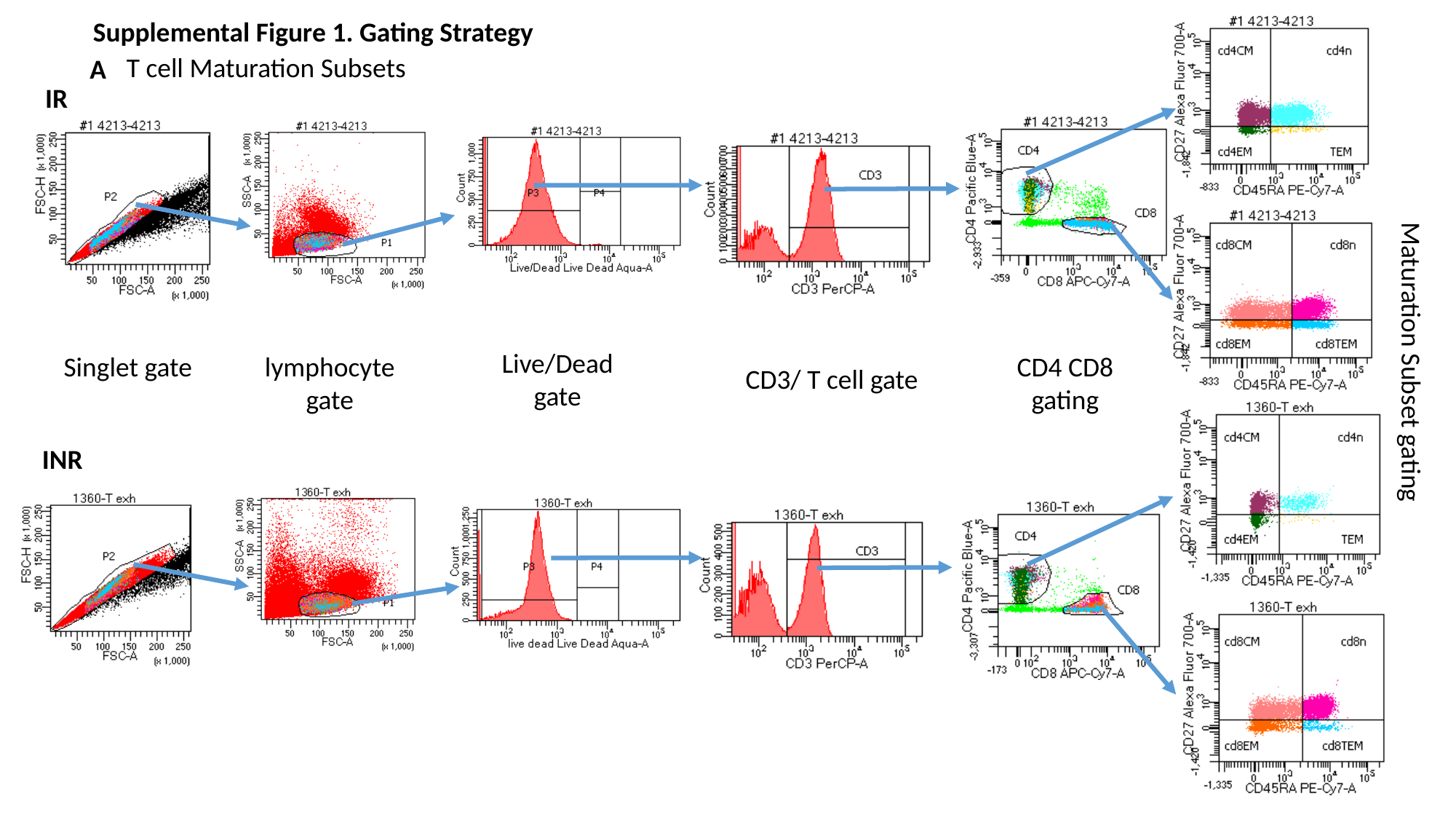

Supplemental Figure 1. Gating Strategy
Live/Dead gate
Maturation Subset gating
Singlet gate
lymphocyte gate
CD4 CD8 gating
CD3/ T cell gate
INR
T cell Maturation Subsets
A
IR

## Slide 2
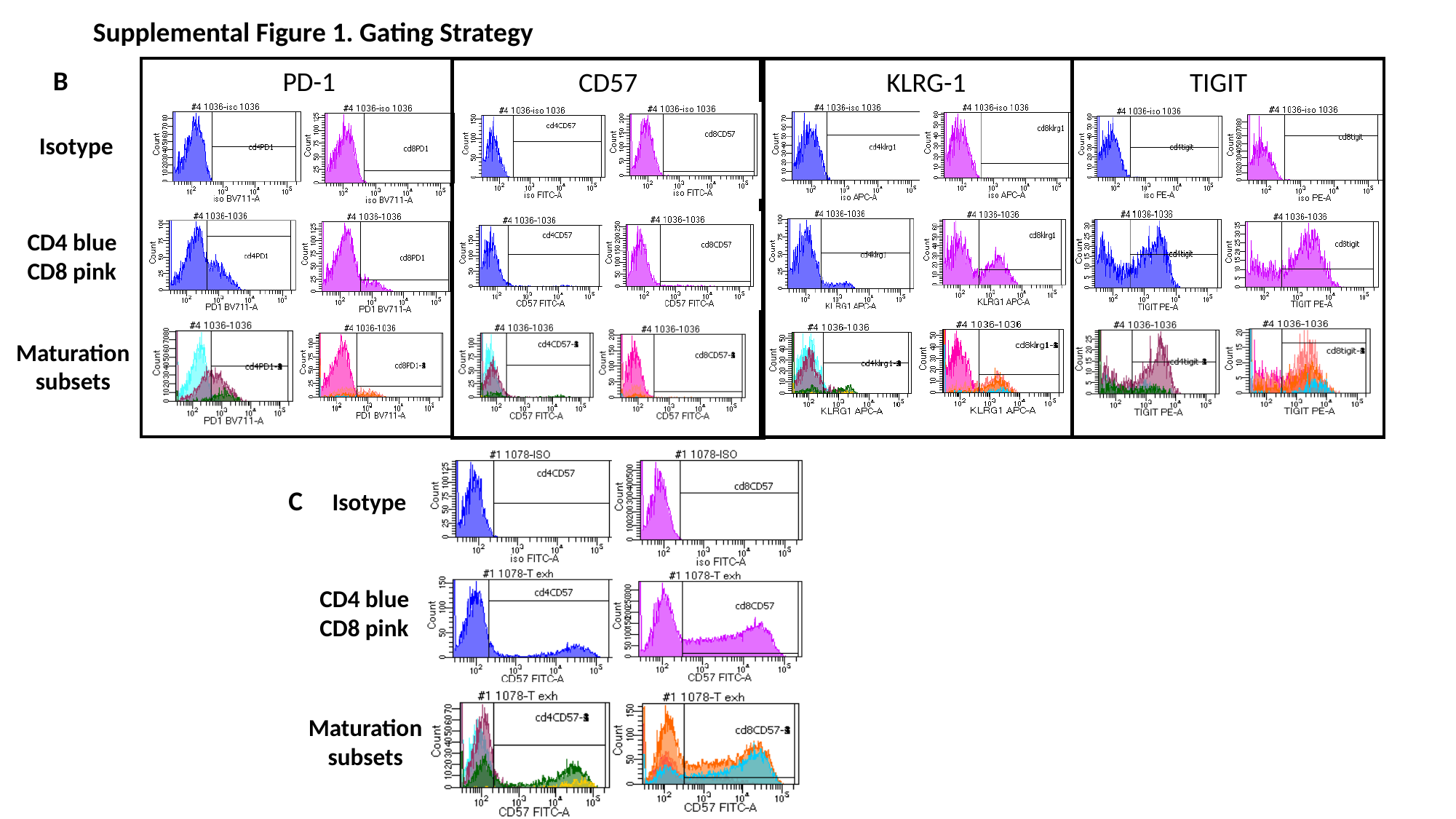

Supplemental Figure 1. Gating Strategy
B
PD-1
CD57
TIGIT
KLRG-1
Isotype
CD4 blue
CD8 pink
Maturation
subsets
C
Isotype
CD4 blue
CD8 pink
Maturation
subsets
