# Supplementary material for: Markers of T Cell Exhaustion and Senescence and Their Relationship to Plasma TGF-β Levels in Treated HIV+ Immune Non-responders
Source: Front Immunol. 2021 Mar 25;12:638010. doi: 10.3389/fimmu.2021.638010 (PMC8044907; doi:10.3389/fimmu.2021.638010)
Supplement: Supplementary Table 1 — The association of plasma levels of IL-6, IP10, TGF-β, and sCD14 with markers of T cell exhaustion and senescence. The correlation of plasma levels of IL-6, IP10, TGFb, sCD14, and sCD163 were compared to the proportions of CD4 and CD8 T cell maturation subsets expressing CD57, PD-1, TIGIT, and KLRG-1 in treated, HIV+ INR, IT, and IR using Spearman's rank order analysis. Only correlations that were significant are shown in table. P and r values are shown for each significant correlation; p = <0.05 considered statistically significant. Gray boxes are significant negative correlations; white boxes are significant positive correlations. [file Table_1.docx]

**Supplemental Table I. The association of plasma levels of IL-6, IP10, TGF-β and sCD14 with markers of T cell exhaustion and senescence**

|  | **Immune Non-responders** | | **Intermediate** | | **Immune Responders** | |
| --- | --- | --- | --- | --- | --- | --- |
| **%** | **CD4** | **CD8** | **CD4** | **CD8** | **CD4** | **CD8** |
| **IL-6** | cd4 EM  CD57  p= 0.030  r= -0.464 |  | cd4 EM TIGIT p= 0.045 r= 0.571 |  | cd4 CM CD57 p= 0.036  r= 0.413 | cd8 EM PD1 p= 0.022  r= -0.448 |
|  | cd4 EM TIGIT p= 0.026  r= 0.474 |  |  |  | cd4 CM PD1 p= 0.014  r= 0.475 |  |
|  |  |  |  |  | cd4 naïve PD1  p= 0.036  r= 0.412 |  |
|  |  |  |  |  | cd4 CM TIGIT p= 0.025  r= 0.440 |  |
| **IP10** | cd4 EM PD1 p= 0.015  r= 0.513 | cd8 CM CD57 p= 0.002  r= 0.629 | cd4 naive CD57 p= 0.042 r= 0.577 | cd8 TEM CD57 p= 0.044 r= -0.627 |  |  |
|  |  | cd8 CM PD1 p= 0.018  r= 0.499 |  |  |  |  |
|  |  | cd8 EM TIGIT p= 0.020  r= 0.491 |  |  |  |  |
| **TGFβ** | cd4 EM CD57 p= 0.002  r= 0.633 | cd8 CM TIGIT p= 0.015  r= -0.510 |  |  | cd4 EM PD1 p= 0.028  r= -0.432 | cd8 naive TIGIT p= 0.034  r= -0.418 |
|  | cd4 EM TIGIT p= 0.034  r= -0.453 | cd8 EM TIGIT p= 0.025  r= -0.476 |  |  | cd4 TEM PD1 p= 0.005  r= -0.700 | cd8 EM TIGIT p= 0.016 r= -0.469 |
|  | cd4 naïve KLRG1  p= 0.010  r= -0.590 |  |  |  | cd4 naïve KLRG-1 p= 0.018  r= -0.461 |  |
| **sCD14** | cd4 CM PD1 p= 0.018  r= -0.499 | cd8 naïve CD57 p= 0.035  r= -0.452 |  | cd8 CM KLRG1 p= 0.031 r= 0.697 |  |  |
|  |  | cd8 naive PD1 p= 0.006  r= -0.570 |  | cd8 EM TIGIT p= 0.043 r= 0.577 |  |  |
|  |  | cd8 CM PD1 p= 0.032  r= -0.459 |  |  |  |  |
|  |  | cd8 naïve KLRG1  p= 0.013  r= -0.547 |  |  |  |  |
|  |  | cd8 TEM KLRG1  p= 0.045  r= -0.478 |  |  |  |  |
